# Evidence for proteolytic cleavage of brevican by the ADAMTSs in the dentate gyrus after excitotoxic lesion of the mouse entorhinal cortex

**DOI:** 10.1186/1471-2202-6-52

**Published:** 2005-08-25

**Authors:** Joanne Mayer, Michelle G Hamel, Paul E Gottschall

**Affiliations:** 1 University of South Florida College of Medicine, Department of Pharmacology and Therapeutics, 12901 Bruce B. Downs Blvd, Tampa, Florida 33612-4799, USA

## Abstract

**Background:**

Brevican is a member of the lectican family of aggregating extracellular matrix (ECM) proteoglycans that bear chondroitin sulfate (CS) chains. It is highly expressed in the central nervous system (CNS) and is thought to stabilize synapses and inhibit neural plasticity and as such, neuritic or synaptic remodeling would be less likely to occur in regions with intact and abundant, lectican-containing, ECM complexes. Neural plasticity may occur more readily when these ECM complexes are broken down by endogenous proteases, the ADAMTSs (adisintegrin and metalloproteinase with thrombospondin motifs), that selectively cleave the lecticans. The purpose of these experiments was to determine whether the production of brevican or the ADAMTS-cleaved fragments of brevican were altered after deafferentation and reinnervation of the dentate gyrus via entorhinal cortex lesion (ECL).

**Results:**

In the C57Bl6J mouse, synaptic density in the molecular layer of the dentate gyrus, as measured by synaptophysin levels in ELISA, was significantly attenuated 2 days (nearly 50% of contralateral) and 7 days after lesion and returned to levels not different from the contralateral region at 30 days. Immunoreactive brevican in immunoblot was elevated 2 days after lesion, whereas there was a significant increase in the proteolytic product at 7, but not 30 days post-lesion. ADAMTS activity, estimated using the ratio of the specific ADAMTS-derived brevican fragment and intact brevican levels was increased at 7 days, but was not different from the contralateral side at 2 or 30 days after deafferentation.

**Conclusion:**

These findings indicate that ADAMTS activity in the dentate outer molecular layer (OML) is elevated during the initial synaptic reinnervation period (7 days after lesion). Therefore, proteolytic processing of brevican appears to be a significant extracellular event in the remodeling of the dentate after EC lesion, and may modulate the process of sprouting and/or synaptogenesis.

## Background

Neurons of the entorhinal cortex (EC) send unidirectional, afferent projections to the hippocampus, where terminals synapse on granule cell dendrites in the outer molecular layer (OML) of the dentate gyrus [1]. Interruption of entorhinal input to the dentate gyrus, by chemical lesion or severing the afferent fibers, causes anterograde degeneration of the axon terminals and stimulates sprouting of viable fibers from this and other neuronal circuits [2-5]. The entorhinal cortex lesion (ECL) has been used as a model of neural plasticity for more than three decades [6]. To identify and innervate a target after injury or during development, growing neuron terminals must traverse through a complex extracellular milieu that consists of soluble factors, cell surface adhesive ligands and an extracellular matrix (ECM) along the way toward its target. The growing terminal samples this milieu, and appropriate protein-protein binding and activation regulates the direction and extent of growth, terminal sprouting, and likely synaptogenesis. For example, certain ECM molecules such as laminin are permissive toward neurite outgrowth, whereas others, such as the highly negatively charged, proteoglycans (PGs) substituted with chondroitin sulfate (CS) (ie. versican, neurocan, aggrecan and brevican, in general, lecticans), inhibit neurite outgrowth on permissive substrates [7-9]. Interestingly, the expression of several lecticans, including brevican is markedly up-regulated during the neural plasticity response that occurs following ECL [10].

The lectican PGs are the most abundant ECM molecules in the adult, uninjured central nervous system (CNS) and of these brevican is the most highly expressed [11,12]. Brevican and other lecticans are found in perineuronal nets and throughout the neuropil and are components of ECM aggregate complexes that are thought to stabilize synapses in neural networks and inhibit neurite outgrowth [13,14]. Most evidence suggests that the CS component in these complexes provides the inhibitory signal toward neurite outgrowth, although the protein core plays a role as well [8,15]. However, proteolytic cleavage of the brevican core protein may "loosen" the aggregated complexes and change the extracellular environment to one that is more permissive for neural plasticity to occur [14,16,17]. A significant proportion of brevican in the adult CNS exists as a fragment formed by proteolytic cleavage of the protein core, suggesting that this mechanism may play a role in experience-dependent and other forms of neural plasticity in the uninjured, adult. In addition, in disorders such as Alzheimer's disease, alterations in the ECM may be related to diminished synaptic plasticity and therefore play a role in cognitive dysfunction.

The metalloproteinases responsible for cleavage of lectican core proteins and the generation of fragments of aggrecan, brevican, and versican have been cloned and belong to a family of proteins, termed the ADAMTSs (adisintegrin and metalloproteinase with thrombospondin motifs), that include glutamyl endopeptidases. ADAMTS1 and ADAMTS4 are prominently expressed in rat brain [16] and cleave the 145 kD intact core protein of brevican into a 55 kD N-terminal, and an 80 kD C-terminal fragment [18,19] (see Fig. 2). The ADAMTS-derived 55 kD fragment may be distinguished from total cleaved fragment (cleaved by other proteinases) using a neoepitope antibody raised against the distinctive C-terminal sequence (EAMESE, murine sequence) of the N-terminal, 55 kD fragment (see Fig. 2, "C") that is generated by glutamyl endopeptidase cleavage. Using this antibody, *in vivo *ADAMTS activity may be estimated by expressing the amount of ADAMTS-derived brevican fragment as a proportion of total intact brevican.

Others have demonstrated changes in the levels and activity of matrix metalloproteinases (MMPs) in the deafferented neuropil after various lesions, however in these experiments, the substrate proteolytically processed by the MMPs was not identified [20,21]. Nonetheless, changes in structure and function that were associated with lesion-induced sprouting were reversed by MMP inhibitors [22]. Lesions produced by systemic injection of kainic acid produce wide-spread neuronal degeneration in the CNS, including the EC and stimulate expression of the MMPs and the ADAMTSs [16,23]. In response to this lesion, the abundance of the neoepitope fragment generated by ADAMTS-cleavage of brevican was markedly elevated in the OML of the dentate gyrus. This increase was preceded by elevated ADAMTS1 and ADAMTS4 mRNA expression in dentate granule neurons [16]. Although these results suggest that proteolytic processing of brevican appears to be a significant extracellular event in the remodeling of the dentate after ECL, because of the widespread neuronal death, it was difficult to associate these two particular endpoints as individual, yet associated events involved in neural plasticity. Thus, we decided to employ the classical ECL model in the C57Bl6 mouse, thereby discretely disrupting synaptic innervation of the molecular layer of the dentate gyrus. Our intention was to observe any altered expression of brevican and the ADAMTSs that may be associated with reinnervation of the injured area 2, 7 and 30 days after lesion.

## Results

### Expression of ADAMTS-derived, brevican fragment

Brevican is an abundantly expressed PG in the CNS that is secreted from astrocytes and neurons as a 145 kD core protein that bears up to three, covalently-linked, CS chains (Fig. 2A). It is also is secreted as a 145 kD core protein without CS chains (Fig. 2B). When cleaved by extracellular glutamyl endopeptidases, the ADAMTSs, a 55 kD N-terminal fragment is formed that contains the unique C-terminal epitope EAMESE (Fig. 2C). Each of these isoforms of brevican was expressed in numerous regions of the CNS. Figure 3A shows a brevican immunoblot of several brain regions from an untreated C57Bl6 mouse. The >145 kD (brevican core plus CS) intact isoform, the 145 kD (brevican core) intact core protein and the generalized 55 kD N-terminal fragment were easily differentiated from one another on Western blot (Fig. 3A) using an antibody that recognizes an epitope in the N-terminal globular domain (Fig. 2). In the mouse, all isoforms of brevican appeared to be more abundant in brain stem and cerebellum compared to other regions that were measured. Panel B shows a blot with the same extracts and probed using the anti-neoepitope EAMESE antibody. This antibody recognized the ADAMTS-derived, C-terminally truncated, 55 kD fragment with the novel C-terminal epitope EAMESE and did not recognize any of the intact, core brevican proteins (Fig. 3B). Thus, given the ability to quantitate the ADAMTS-derived brevican fragment and its intact substrates using immunoblot, regional *in vivo *ADAMTS activity may be estimated.

### Deafferentation and neural plasticity in the dentate gyrus

To validate a complete deafferentation of this synapse of the perforant path, from the EC to the molecular layer of the dentate gyrus, animals were injected with ibotenic acid into the lateral EC, perfused with fixative, the brain sectioned, and immunostained for brevican. Brevican is a marker expressed by reactive glia in response to brain injury [24]. Thus, after lesion, increased deposition of brevican would be expected in the ipsilateral EC. Brevican immunoreactivity was elevated at the lesion site (Figure 4B) compared to the contralateral, non-lesioned EC (Figure 4A).

Since an endpoint in this experiment is the reinnervation and sprouting of neurites and the formation of new synapses in the OML of the dentate gyrus, synaptophysin immunoreactivity, a vesicular pre-synaptic marker, was examined two, seven, and thirty days post-lesion. On the contralateral side and in unlesioned mice, a tri-laminar synaptophysin staining pattern was observed that outlines the inner, middle and OMLs (Fig. 5B) of the dentate gyrus. Two days after ECL, there was a marked decline in synaptophysin immunoreactivity in the OML on the ipsilateral side of the dentate (Fig. 5A), that was reflected by a loss of the typical tri-laminar pattern of synaptophysin staining (Fig. 5B) with only the inner molecular layer clearly distinguishable. Seven days after ECL, synaptophysin immunoreactivity in the OML remained markedly reduced, although the pattern was similar to the contralateral side at 30 days (data not shown).

**Figure 1:**
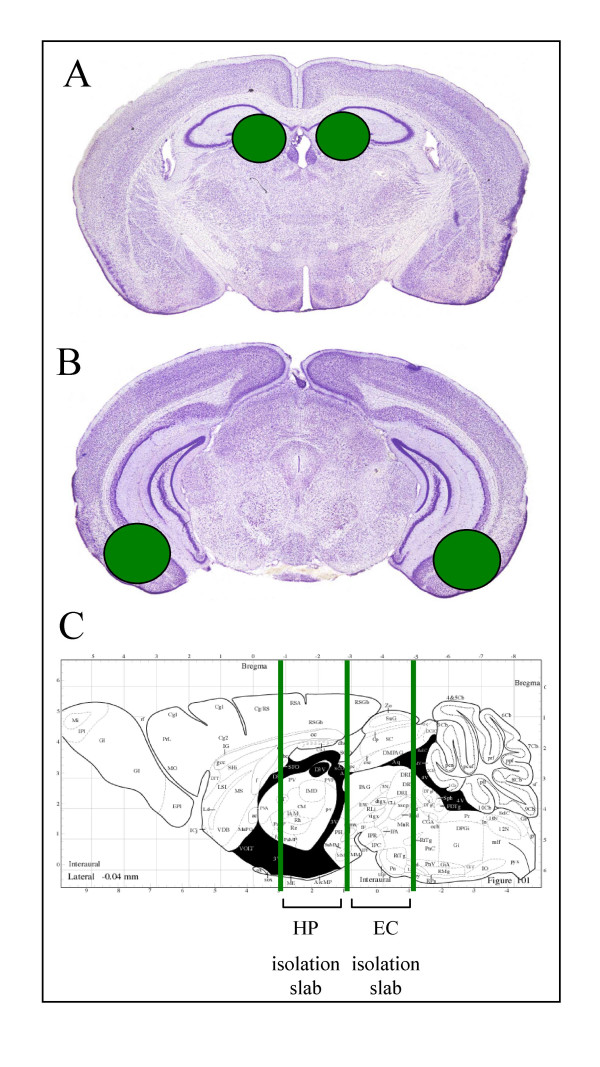
**Schematic representation of the micro-dissection of dentate gyrus and entorhinal cortex: **This procedure was developed to isolate dentate gyrus and entorhinal cortex tissue from different rostral-caudal slabs in fresh-frozen mouse brain. A: Coronal section of mouse brain showing bore holes left from blunt-ended, 22 gauge needle punch (green) in the ipsilateral and contralateral dentate gyrus. B: Coronal section of mouse brain (2 mm caudal from section in "A") showing bore holes left from blunt-ended, 18 gauge needle punch (green) in the ipsilateral and contralateral entorhinal cortex. C: Sagittal view of 2 mm slices used for the collection of dentate gyrus (A) and entorhinal cortex (B). Diagrams adapted from 'The Mouse Brain' [46].

**Figure 2:**
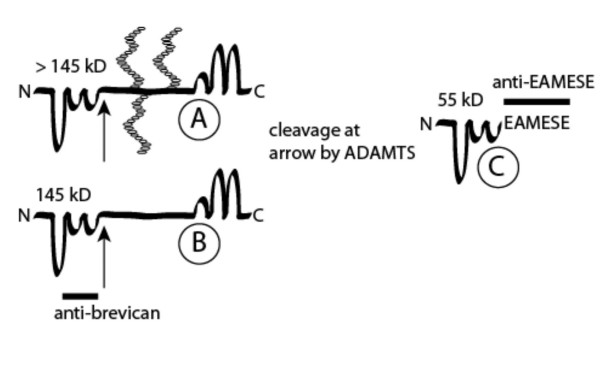
**Schematic representation of brevican cleavage by the glutamyl-endopepidases, the ADAMTSs: **A: Secreted brevican core protein that bears chondroitin sulfate chains (MW > 145 kD). B: Secreted brevican core protein without chondroitin sulfate side chains (MW = 145 kD). C: When cleaved by extracellular glutamyl endopeptidases, the ADAMTSs, an N-terminal fragment of brevican (MW = 55 kD) is formed that contains the unique C-terminal epitope EAMESE. The anti-brevican antibody recognizes all three isoforms of brevican, whereas anti-EAMESE antibody selectively recognizes the ADAMTS-derived proteolytic fragment of brevican and not the parent protein or other isoforms.

**Figure 3:**
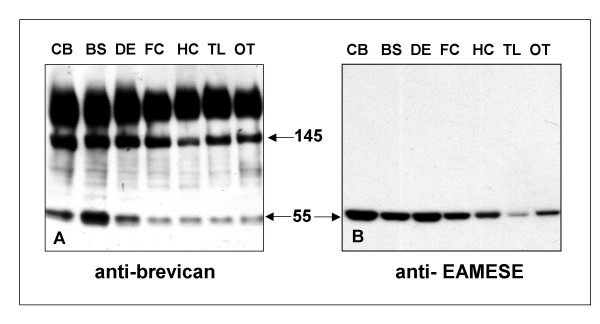
**Brevican and EAMESE Western blotting in regions of mouse brain: **Panel A shows an immunoblot containing extracts from various regions of mouse brain and probed with anti-brevican antibody. Immunoreactive bands are present at >145 kD (high molecular weight smear), 145 kD core protein, and the generalized 55 kD fragment. Panel B contains a membrane with the same samples probed with anti-EAMESE. This antibody recognized the specific ADAMTS-derived 55 kD fragment of brevican. CB = cerebellum, BS = brain stem, DE = diencephalon, HC = hippocampus, FC = frontal cortex, TL = temporal lobe, and OT = olfactory tubercle.

**Figure 4:**
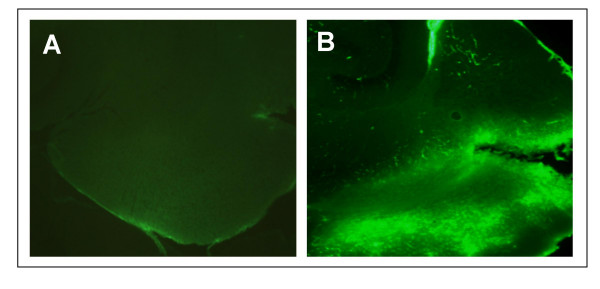
**Alterations in brevican levels in lesioned entorhinal cortex: **Horizontal sections of entorhinal cortex immunostained with anti-brevican antibody collected two days after entorhinal cortex lesion. A: Contralateral entorhinal cortex showed little immunoreactive signal. B: Ipsilateral entorhinal cortex showed abundant anti-brevican staining in the lateral region.

**Figure 5:**
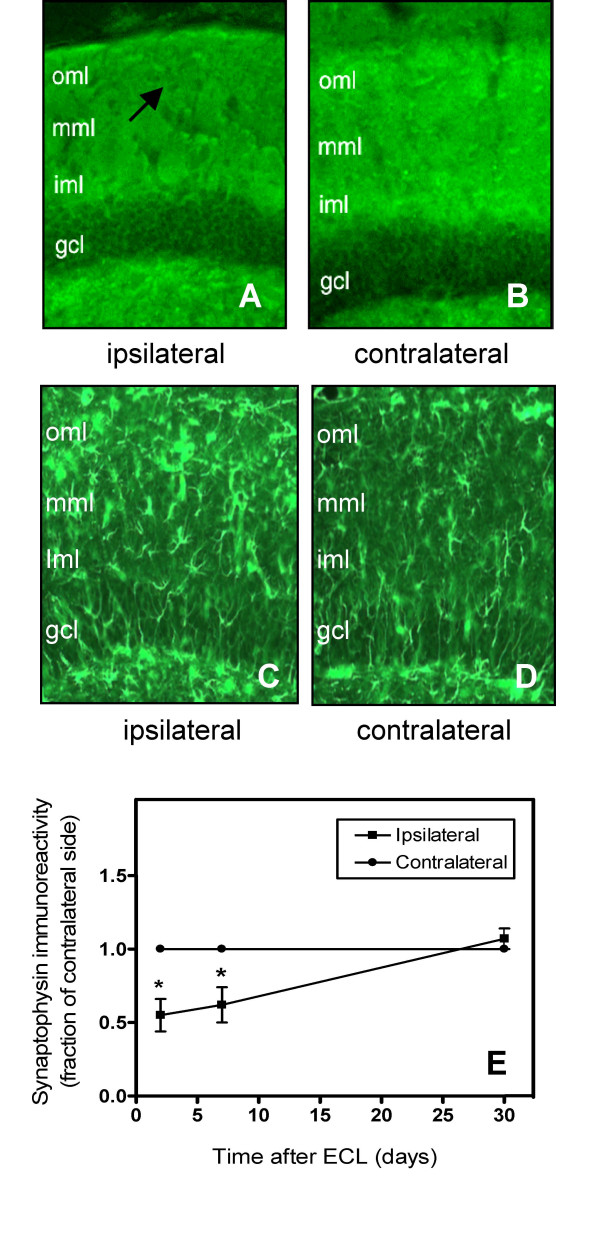
**Denervation of the outer molecular layer of the dentate gyrus after entorhinal cortex lesion: **A and B: Synaptophysin immunoreactivity in the molecular layer of the dentate gyrus two days after ECL A: ipsilateral dentate, B: contralateral dentate. Note diminished immunostaining and loss of tri-laminar pattern in the OML after ECL. C and D: GFAP immunoreactivity in the molecular layer of the dentate gyrus seven days after ECL. Note increased astroglial expression of GFAP observed after deafferentation of the ipsilateral (C), but not contralateral (D), OML. E: Synaptophysin levels in extracts from fresh micro-dissected (needle punch, see Fig. 1) dentate gyrus tissue as measured by ELISA. Synaptophysin immunoreactivity was reduced two and seven days post-lesion. The ipsilateral side was not different from contralateral dentate thirty days after lesion. oml = outer molecular layer, mml = middle molecular layer, iml = inner molecular layer, and gcl = granule cell layer.

In response to injury and the deafferentation of synapses, there is an activation of astrocytes in the OML [25]. Tissue immunostained with the astrocytic marker GFAP (glial fibrillary astrocytic protein), showed increased staining selectively in the OML of the dentate seven days after ECL (Fig. 5C) compared to the contralateral side (Fig. 5D). Although these markers may be sufficient to identify some level of diminished innervation to the OML, a synaptophysin ELISA and a specific method to isolate the tissue of the dentate gyrus (Fig. 1) was developed to better quantitate this loss.

Two days after lesion, synaptophysin levels as detected by ELISA, declined by 46% in the ipsilateral dentate compared to the contralateral side (p = 0.039) (Fig. 5E). This trend was sustained at seven days post-lesion, and synaptophysin levels were 41% of the contralateral value (p = 0.033) (Fig. 5E). However, when tissue was examined thirty days post-lesion, synaptophysin concentrations were not different from the control side. This observation shows there is a complete reinnervation of the OML after ECL (Fig. 5E).

The decline in the level of synaptophysin concentrations in the tissue from the "dentate punch" on the ipsilateral side compared to the contralateral side indicates that there was denervation of cortical input to the dentate molecular layer. The question of interest was, however, were there associated changes in the abundance and proteolysis of brevican in this region of neural plasticity?

### Abundance and proteolysis of brevican after ECL

**Figure 6:**
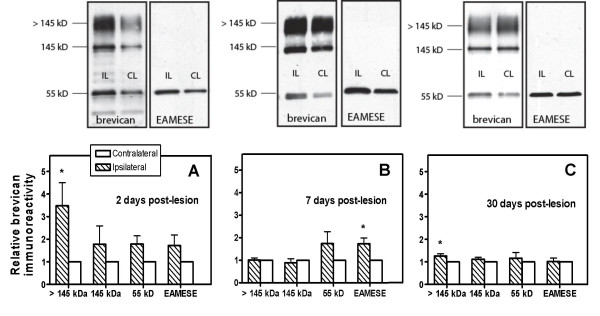
**Brevican immunoreactivity in the dentate gyrus after entorhinal cortex lesion: **Brevican immunoreactivity in the contralateral (CL) and ipsilateral (IL) dentate gyrus of mice that had undergone ECL two, seven, and thirty days earlier. Optical density was measured on the Western blots and data from the ipsilateral side was expressed as a fraction of the contralateral dentate. A: At two days, the CS-containing, >145 kD brevican was significantly elevated on the lesion side compared to the contralateral side although all fragments showed a trend for increase. B: At seven days, there was a significant elevation of the ADAMTS-derived EAMESE fragment of brevican and a trend for an increase in 55 kD fragment. C: At thirty days, brevican isoforms were not different from the control side, except that the >145 kD core protein was slightly elevated.

Brevican and its various isoforms were identified in immunoblots from isolated dentate gyrus tissue obtained from mice two, seven and thirty days after ECL. At two days post-lesion (Figure 6A), there was a 3-fold increase in the glycosaminoglycan (GAG)-containing form of brevican (p = 0.047) in the dentate on the denervated side, and a trend for an increase in all the isoforms of brevican on this side. At seven days after insult, a time thought to be the initial synaptic reorganization period after ECL [26], intact brevican had returned to contralateral levels, but there was a significant, almost 2-fold elevation in the ADAMTS-derived fragment of brevican in the dentate (p = 0.030). In addition, there was a trend for an increase in the generalized 55 kD fragment of brevican (Fig. 6B) at this time point. Thirty days after undergoing surgery, all of the forms of brevican had returned to levels not different from the contralateral side; however, a slight increase in the GAG-containing, intact isoform was seen at this time point (Fig. 6C).

**Figure 7:**
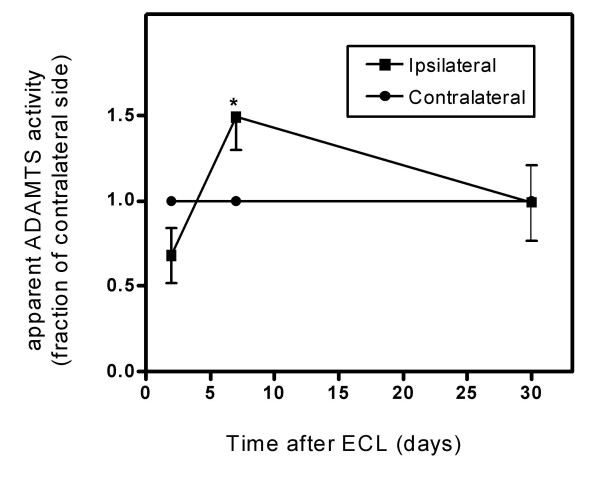
**Apparent ADAMTS activity: **Apparent activity in the dentate gyrus of mice after entorhinal cortex lesion as measured by the ratio of the optical density of the ADAMTS-derived EAMESE fragment and the sum of the densities of the brevican core protein isoforms (>145 kD + 145 kD). Mean level of apparent activity was calculated at two, seven and thirty days after lesion. At two days after lesion, ADAMTS activity declined, whereas at seven days a significant 47% increase in ADAMTS activity was observed compared to the contralateral ADAMTS activity. Levels on the lesioned side were not different from the non-lesioned dentate in tissue collected thirty days after lesion.

To estimate apparent ADAMTS activity *in vivo*, the density of the EAMESE (55 kD) immunoreactive fragment in Western blot was divided by the densitometric level of intact full-length and core protein brevican isoforms (>145 kD + 145 kD) (Fig. 7). Two days post-ECL there was a decrease in apparent ADAMTS activity in the ipsilateral dentate gyrus, however, at the seven day critical reinnervation period, ADAMTS activity was increased almost 50%. This observation is supported by the significant increase in the ADAMTS-derived, 55 kD fragment at this time point. By thirty days post-lesion, the activity was not different from the contralateral side. Thus, this data suggests that the ADAMTSs and the catabolism of brevican may play a prominent role in neural plasticity in the dentate gyrus after lesion of the EC in mice.

## Discussion

The purpose of this study was to determine whether the catabolism of brevican is involved in mechanisms of neural plasticity in the hippocampus, and to accomplish this, synaptic input to the OML of the dentate gyrus was denervated by excitotoxic lesion in the lateral EC. Two days after lesion, synaptic input into the OML was significantly reduced and this was accompanied by an increase in the production of full length, intact brevican. At seven days, while brevican levels returned to baseline, a significant increase in the ADAMTS-derived, C-terminally truncated, brevican fragment was observed during this initial, sprouting and reinnervation period. This implies that there was an increase in ADAMTS activity in the OML during the highly plastic, regenerative phase. However at thirty days post-lesion, there was complete reinnervation of the OML on the ipsilateral side, as synaptic density, brevican and ADAMTS activity were not different from the contralateral side at this time point. These results indicate that the ADAMTSs and their substrate, brevican, that is abundant in the CNS, have a regulatory function in neural plasticity and support earlier data that had demonstrated important actions for the ADAMTSs in plasticity after seizure-induced hippocampal lesion [16].

Previous studies have examined the role of matrix-altering proteases in synaptic plasticity after CNS lesion. The expression of the matrix metalloproteinases (MMPs), MMP-9 and MMP-2, have been shown to be increased in various regions of the hippocampus after seizure-induced lesion [21,23,27,28]. MMP-3 concentrations were elevated in the molecular layer of the dentate after traumatic brain injury [29]. More specifically after ECL, administration of a non-selective MMP inhibitor was able to diminish sprouting and synaptogenesis in the dentate OML [22], suggesting a direct proteolytic role for the MMPs in this process. In adults, most MMP expression and activity is low and maintained throughout adulthood. After injury and during the recovery and regenerative phase, however, there is increased activity of MMPs derived from glia and neurons that is thought to facilitate axonal reinnervation, sprouting and/or synaptogenesis. Nonetheless, the mechanism(s) of action of the MMPs and the potential substrates on which they act to promote neural plasticity have yet to be determined in these models. More recently, the activity and expression of the PG-degrading, ADAMTSs have been shown to be elevated in the OML after kainite-induced lesion. In contrast to the absence of a defined substrate for the MMPs, a selective ADAMTS-derived, brevican fragment was localized to the OML after seizure-induced lesion in the rat [16]. In the present study, a similar ADAMTS-derived brevican fragment was localized to the OML of the mouse after discrete denervation of the perforant path, suggesting a critical role in neural plasticity for the proteolytic turnover of brevican. Thus, the ability to localize and quantitate the ADAMTS specific, proteolytic product of brevican provides a means to indirectly estimate ADAMTS activity during times of neural plasticity and synaptogenesis.

The expression of brevican was shown previously to be up-regulated in the OML, the area of denervation after ECL in the rat [10], however, in contrast to the transient production observed here in the mouse, expression of immunoreactive brevican remained elevated compared to the non-lesioned side for almost 6 months after injury. Neurocan is a lectican that is expressed at high levels during early development but it was found to be up-regulated and synthesized by astrocytes in the OML after ECL. It was suggested that neurocan and possibly brevican may act to maintain the boundary of the denervated dentate after ECL [30], yet these complex molecules may be multifunctional during periods of neural plasticity. Each of the lecticans, exhibit a characteristic pattern of expression during development, with neurocan and versican V1 highly expressed in the brain of the fetus and neonate, whereas aggrecan, versican V2 and brevican increase expression during the period of synaptic stabilization in the adult and expression remains high throughout adulthood [13,31]. Each of the lecticans is thought to bind to tenascin R and hyaluronic acid (with varying affinities) forming a multi-molecular lattice of ECM [14]. It may be that proteolytic cleavage of the lectican loosens the lattice to promote neurite growth and synaptogenesis. Classically, the highly negatively charged CS chains on the lecticans inhibits neurite outgrowth, but proteolytic cleavage of the core protein may allow more movement of these chains and actually promote plasticity of neurons. This is a testable notion and preliminary data indicates that the ADAMTSs promote neurite outgrowth and other measures of neural plasticity *in vitro *(our unpublished observations).

The projection from the EC to the hippocampus is called the perforant path [32], and is thought to be involved in long-term potentiation and learning and memory [33-35]. The ECL model to study neural and synaptic plasticity denervates up to 80% of the input to the outer two-thirds of the molecular layer of the dentate gyrus [36], and due to sprouting of surviving fiber systems will reinnervate nearly fully. This model was developed more than thirty years ago in the rat [37], yet it has been only relatively recently that the technique was employed in mice to take advantage of transgenic models [38,39]. Surprisingly, there are some differences in the projections from the EC to the hippocampal formation between rats and mice [40]. For example, input to the dentate molecular layer from the contralateral EC is absent in the mouse, yet these contralateral fibers are responsible for much of the sprouting after ECL in the rat. In addition, the width of the inner molecular layer, that contains associational-commissural fibers, is thinner in the mouse than in the rat, causing an increase in the relative width occupied by the middle and OMLs, layers innervated mainly by EC fibers. In the mouse, the middle and OML occupy closer to four-fifths of the total, rather than two-thirds as seen in the rat. Moreover, three layers can be clearly differentiated in an untreated mouse, but not in a rat, using synaptophysin immunohistochemistry [40], and following ECL this laminar feature is lost (Fig. 5B). Synaptophysin immunochemistry has been one of the more common techniques, among many, to quantitate the loss and reinnervation of input into the ipsilateral molecular layer of the dentate gyrus after unilateral ECL [41]. Optical density of the synaptophysin signal in the contralateral OML is measured using the sum (or average) of the gray levels of the pixels in this region, and this value is used as a "normal" value to the ipsilateral side. However, with this technique, the ipsilateral dentate usually shows only a 10–30% reduction in signal compared to the contralateral side at seven days after lesion, a time when sprouting has begun [42]. Clearly this absolute value does not reflect the extent to which fibers are actually lost in the OML after ECL. Thus, we decided to develop a fresh tissue, needle punch dissection technique that could be limited to the dentate gyrus in the mouse. This way, biochemical assays could be conducted on the tissue to measure overall synaptophysin immunoreactivity by ELISA. Using this method, at two and seven days after lesion, there was greater than a 40% reduction in synaptophysin levels in the OML, a value which at least may closer reflect the absolute loss of fibers after ECL. The major disadvantage of this method is that the dissected tissue also includes the granule layer and the hilus of the dentate, regions where input is not lost after ECL. At the same time using this technique, tissue containing the lesion itself may be collected and assayed biochemically or processed for histochemistry to monitor the extent of the lesion in the EC.

The present results suggest that the lecticans and the proteases that cleave the lecticans play a regulatory role in neural plasticity after ECL. There are several potential mechanisms by which this substrate – protease pair may modulate neural plasticity, one of which was described above. Significant changes were observed in the abundance of the different isoforms of brevican including the expression of the C-terminally-truncated ADAMTS-derived brevican fragment during plastic events in the hippocampus. A genetic approach to study the individual lecticans and ADAMTSs could reveal the individual contributions for each of the molecules involved in neural plasticity after ECL, however, there is considerable redundancy among these molecules. For example, there appears to be a compensatory increase in the expression of neurocan in the brain of the brevican null mouse. [43] In addition, several of the ADAMTSs exhibit proteoglycanase activity, and of these, at least ADAMTS1, 4 and 9 appear to be expressed in the nervous system (unpublished observations). Whether there are compensatory changes in the expression of any of these molecules in the brain in ADAMTS null mice remains to be determined. Nonetheless, significant protective effects toward arthritic changes were demonstrated just recently in a single mutant, the ADAMTS5 null mouse [44]. Should it turn out that these proteases play a significant role in plasticity related mechanisms in the nervous system, it will be interesting to examine how removing this regulatory action will impact development, sprouting after lesion, learning and memory and other plasticity related mechanisms in the adult.

## Conclusion

Growth, sprouting and targeting in neural plasticity of an ECL model may involve extracellular cues whose expression and/or secretion is altered following the lesion. One of these cues is the extracellular PG, brevican. Here we have demonstrated changes in brevican expression and turnover after ECL that are associated with the loss and time-dependent reinnervation of the OML of the dentate gyrus.

## Methods

### Animals

All animal procedures described here were approved by the Institutional Animal Care and Use Committee at the University of South Florida. Sixty-two adult male C57Bl6 mice (23 g – 27 g; Harlan, Indianapolis, IN), 12 weeks of age were housed under a 12 h light cycle with regulated temperature and humidity. Mice were housed 3 to 4 per cage and had free access to food and water. Following ECL surgery, the animals were housed individually. Brains from control mice (n = 4) and lesioned mice surviving for 2 days (n = 6), 7 days (n = 5) and 30 days (n = 5) after the lesion were perfusion fixed and collected for immunohistochemistry. Tissue extracts of dentate gyrus and EC lesioned animals collected 2 days (n = 6), 7 days (n = 5) and 30 days (n = 6) after surgery were used in Western blot and biochemical immunoassays. For those animals that received a unilateral ECL, the contralateral, non-lesioned hemisphere was considered the control for immunohistochemistry and biochemical analysis.

### Surgical procedures – the entorhinal cortex lesion (ECL)

Surgeries were performed using isofluorane/oxygen mixed gas anesthesia. Once deeply anesthetized, animals were placed into the stereotaxic apparatus. A hole was drilled in the skull of the right hemisphere to allow for needle penetration. The right, lateral EC of mice was unilaterally lesioned by lowering a needle attached to a Hamilton syringe (#701N) filled with ibotenic acid through the hole in the skull to the coordinates AP = 4.72 mm, L = 3.75 mm and DV = 4.70 mm using bregma as a reference and oriented 17° rostral-caudal [45]. One μl of the neurotoxin, ibotenic acid was injected into the lateral EC at a rate of 0.1 μl every 30 seconds, with the needle remaining in place for an additional minute at the end of the 5 min injection period to allow for complete diffusion of the drug into the lateral EC. Neurons of the lateral EC project preferentially to the septal dentate gyrus. The needle was removed, bone wax used to cover the skull hole, and the animal was allowed to recover on a heating pad after which the animal was returned to a new cage and housed individually. At 2, 7 and 30 days after lesion, mice were injected with an overdose of Nembutal (pentobarbital) for deep anesthesia, perfused transcardially with cold phosphate-buffered saline (pH 7.4) followed by cold 4% paraformaldehyde fixative diluted in 0.1 M phosphate buffer (pH 7.4). The brain was dissected from the skull, post-fixed in 4% paraformaldehyde overnight at 4°C, cryoprotected with consecutive solutions of 15 and 30% sucrose until completely infused, and the cryoprotected brain was sectioned on a cryostat at 30 μm. The extent and magnitude of the lesion in the EC was verified by cresyl violet and brevican staining. Synaptophysin and GFAP immunohistochemistry, using horseradish peroxidase amplification and diaminobenzidine as a chromogen, verified the loss of terminals in the molecular layer of the dentate gyrus.

### Region isolation method

A method was developed to isolate dentate gyrus and EC tissue in mouse brain. This method eliminates the collection of much of the surrounding tissue that was unaffected by ECL. The dentate gyrus was subjected to synaptophysin ELISA as a measure of synaptic loss in the molecular layer. The EC underwent Western blot for PSD (post-synaptic density) 95 to quantitate the magnitude of the lesion. In addition, brevican and EAMESE Western blot was conducted on dentate gyrus tissue. Animals were completely anesthetized with a lethal dose of Nembutal (pentobarbital, Abbott Laboratories, North Chicago, IL), the brain was quickly removed and placed on a glass microscope slide, and a mm ruler was placed adjacent to the brain to assist in measuring the thickness of slices. Whole brain was dissected and frozen on a flat slab of dry ice for about 5 minutes (a point at which the brain surface is no longer shiny), being careful not to over-freeze the tissue, but to reach a temperature at which the tissue can be easily and efficiently cut into 2 mm coronal slabs. Two 2 mm coronal slabs are cut by referencing the most caudal of cerebral cortex as a land mark, making a coronal cut, moving rostrally 2 mm and making another cut to obtain the first slab for the EC isolation. A third cut was made 2 mm rostral from the previous slice and this slab contained the septal hippocampus and was used to isolate the dentate gyrus. The 2 mm hippocampal slab (outlined in Fig. 1C) was placed on a glass slide, and the dentate gyrus was localized using a stereomicroscope. The dentate was punctured with a blunt-ended 22 gauge needle (Fig. 1A) using the medial point of the wings as a landmark. A blunt-ended 18 gauge needle was used in the same manner to puncture and collect the EC from a 2 mm slab caudal to the slab used to collect the dentate gyrus (Fig. 1B). The frozen tissues were immediately expelled from the needle by attaching an air-loaded syringe to the needle and forcing the tissue into the bottom of a microfuge tube. The dentate gyrus and EC tissues were then homogenized in extraction buffer (20 nM Tris-HCL pH = 7.4, 5 mM EDTA, 1% Triton-X-100, & 1:100 protease inhibitor; 20 μl for dentate gyrus and 40 μl for EC) using three cycles of 2 min of 4°C incubation and 30 sec of vortex. The solubilized extract was centrifuged at 8000 rpm in a refrigerated microfuge for 3 min, the supernatant collected and frozen for later use in Western blotting and ELISA analysis.

### Immunohistochemistry

Mice were anesthetized with an overdose of Nembutal (pentobarbital, Abbott Laboratories, North Chicago, IL) and their brains were fixed via standard cardiac perfusion methods. Briefly, the animal was cleared with phosphate buffered saline (PBS; pH 7.4) and fixed with 4% paraformaldehyde in 0.1 M phosphate buffer (PB; pH 7.4). The brains were post-fixed overnight in the same fixative; cryoprotected with 15% sucrose (in 0.1 M PB) followed by a 30% sucrose solution for 24 h each. Whole brain was frozen with mounting medium and cut into 30 μm coronal sections using a cryostat. Free-floating sections were washed with PBS, placed in a blocking/permeabilization solution (10% normal goat serum; 3% 1 M Lysine, 3% Triton-X) for 1 h, washed with PBS and incubated with primary antibody overnight at 4°C. For detection of the antigen, sections were washed and incubated in secondary antibody solution (anti-rabbit or anti-mouse IgG conjugated to Alexa-Fluor 488 or 594, Molecular Probes, Eugene, OR) for 1 h. The sections were washed for 15 minutes, wet mounted onto Fisher SuperFrost Plus glass sides and coverslipped with VectaShield mounting medium (Vector Labs, Burlingame, CA).

The primary antibodies or probes used in these experiments were: mouse anti-brevican, raised against the G1 domain of brevican (BD Transduction Labs, San Jose, CA); rabbit anti-EAMESE, the ADAMTS-derived neoepitope of brevican developed in our lab; rabbit anti-glial fibrillary acidic protein (GFAP, DAKO, Carpinteria, CA); and rabbit anti-synaptophysin (DAKO).

### Western blotting

Dentate gryus or EC extracts were loaded (equal amounts of protein and 2 × sample buffer) on to 4–20% polyacrylamide gels (Invitrogen, Carlsbad, CA) and subjected to SDS-PAGE. Protein was transferred to a polyvinylidine difluoride membrane (PVDF, Immobilon, Millipore, Billerica, MA) and the membrane was blocked with 5% milk in PBS. Membranes were probed with primary antibodies against brevican (1:1000), EAM (1:500), and secondary anti-rabbit or anti-mouse IgG conjugated to horse radish peroxidase (Chemicon, Temecula, CA). Antigens were visualized using a chemiluminescence developing system (SuperSignal, Pierce, Rockford, IL).

### Antibody generation

A rabbit antibody raised against the brevican neoepitope on the 55 kD N-terminal fragment derived from glutamyl endopeptidase activity of the ADAMTSs was generated by Sigma-Genosys (St. Louis, MO) and purified in our laboratory. The novel C-terminal sequence "QEAMESE" from the mouse was the neoepitope and the peptide used for antibody generation contained a glycine spacer "GGGQEAMESE". This peptide was synthesized by Sigma-Genosys, conjugated to keyhole limpet hemocyanin at the N-terminus, and rabbits were subjected to standard immunization protocols. Serum collected after the 5th booster was titered against the peptide using a solid-phase system and specific antibody was purified using peptide affinity chromatography. Interestingly, antibody raised against the rat epitope "QEAVESE" did not recognize the mouse, ADAMTS-derived, "QEAMESE" epitope. On Western blot, the antibody against the mouse fragment recognized a single band at 55 kD in extracts from mouse brain and did not cross react with the intact brevican core protein.

## Authors' contributions

JM performed the surgical procedures and carried out the immunoassays, statistical analysis and drafted the manuscript. MH was involved in the development of the synaptophysin ELISA. PG conceived of the study, participated in its design and coordination, performed statistical analysis and helped draft the manuscript. All authors read and approved the final manuscript.
